# Comparison of the maximum hand-generated torque by professors and postgraduate dental students for tightening the abutment screws of dental implants

**DOI:** 10.15171/joddd.2018.029

**Published:** 2018-09-18

**Authors:** Feridoun Parnia, Javad Yazdani, Parisa Fakour, Farhang Mahboub, Seyyed Mahdi Vahid Pakdel

**Affiliations:** ^1^Dental and Periodontal Research Center, Tabriz University of Medical Sciences, Tabriz, Iran; ^2^Department of Prosthodontics, Tabriz University of Medical Sciences, Tabriz, Iran; ^3^Department of Oral and Maxillofacial Surgery, Faculty of Dentistry, Tabriz University of Medical Sciences, Tabriz, Iran; ^4^Private Practice, Tabriz, Iran

**Keywords:** Torque Force, abutment screw, loosening

## Abstract

***Background.*** Dental implants are utilized with an ever-increasing rate. One of the causes of abutment screw loosening has been identified as inadequate preload. The objective behind this study was to compare the maximum hand-generated torque for tightening abutment screws by professors and postgraduate dental students using a digital torquemeter with 0.1 N/cm precision.

***Methods.*** In a laboratory study conducted in Dental Implant Department of Faculty of Dentistry, Tabriz University of Medical Sciences, the maximum hand-generated torque for tightening abutment screws by professors and postgraduate dental students was investigated, using a digital torquemeter with 0.1 N/cm precision.

***Results.*** The participants consisted of 36 (41.9%) females and 50 (58.1%) males, totaling 86 participants, of whom 45 (46.87%) and 41 (53.13%) were university professors and postgraduate dental students, respectively. The mean age of the participants was 33.4±10.2 years with an age range of 25‒60 years; 50 (58.1%) participants were in the 25‒34-year, 23 (26.7%) in the 35‒47-year, and 13 (15.1%) in the 48‒60-year age range. The mean age of professors and postgraduate dental students was 41±8.3 and 25.1±3.3 years, respectively. The means of maximum torques generated by female and male professors were 14.3±3 and 20.8±4.2, respectively. The means of maximum torques generated by female and male postgraduate dental students were 14.7±3.4 and 18.7±4.3, respectively. Statistical analyses showed no significant differences between the mean maximum torques generated by professors and postgraduate dental students (P=0.051).

***Conclusion.*** In the present study, the mean maximum torque generated by professors was higher than that generated by postgraduate dental students. However, the difference was not statistically significant. The mean maximum torque generated by male subjects was significantly higher. No interaction was seen between the studied groups and sex. However, there was a statistically significant difference between the mean maximum torques generated in different age ranges; i.e., the maximum torque generated in the 25‒34-year age range was lower than that in the other two age groups. Finally, the effect of age range on the mean maximum torque was similar in both groups.

## Introduction

Currently, there is widespread use of dental implants^1^ and use of these implants in completely or partially edentulous patients has been associated with long-term clinical success.^2^ The success of dental implants has a direct relationship with observation of proper surgical and prosthetic protocols.^2^ Despite the fact that dental implant treatments exhibit high success rates, prosthetic and surgical complications in implant-supported prostheses are not uncommon.^3^ These complications might include intraoperative problems, bone loss, peri-implantitis, esthetic and phonetic problems and finally the prosthetic biomechanical complications.^4^ Prosthetic complications might include veneer fractures, abutment screw loosening, screw fracture, and fractures of the metallic framework and the implant itself,^5,6^ of which the abutment screw loosening is the most common and the most important problem.^2-6^

Some of the etiologic factors for abutment screw loosening are insufficient preload, the improper position of the implant, inappropriate occlusal profile or the anatomy of the crown, variations in the dimensions of the hex, inappropriate adaptation of implant components, incorrect design of the screw, occlusal overload and inappropriate antirotationfeatures.^7-9^ The recommended force for tightening of the abutment screw is 20‒30 N/cm.^2^ Based on the results of various studies, individuals produce a wide range of torque, depending on their individual characteristics.^1-12^

Therefore, the present study was designed to determine and compare the maximum hand-generated torque for tightening of the abutment screw by professors and undergraduate postgraduate dental students in the Department of Prosthodontics, Tabriz Faculty of Dentistry.

## Methods

**Figure 1:**
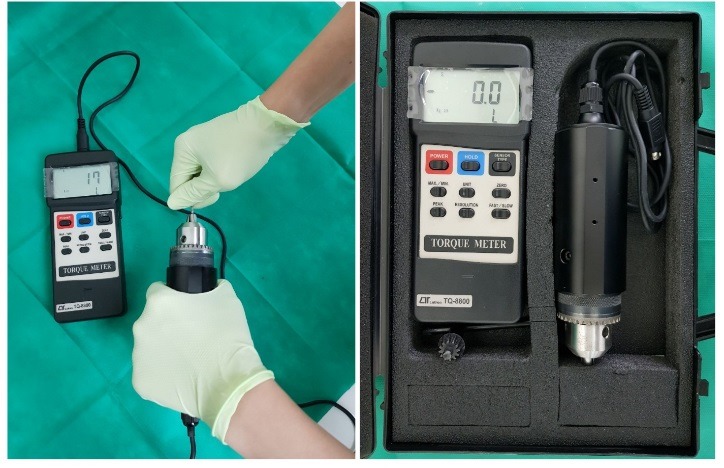
The maximum hand-generated torque for tightening of abutment screws by professors and postgraduate dental students was measured with the use of a digital torquemeter (Iotron, TQ8800, Taiwan) accurate to 0.1 N/cm.

In the present in vitro study, carried out in the Department of Prosthodontics, Faculty of Dentistry, Tabriz University of Medical Sciences, the maximum hand-generated torque for tightening of abutment screws by professors and postgraduate dental students was measured with the use of a digital torquemeter (Iotron, TQ8800, Taiwan, Figure 1) accurate to 0.1 N/cm.

The subjects consisted of two groups, including professors and the last-year undergraduate postgraduate dental students in the Department of Prosthodontics in 2014.

All the professors and postgraduate dental students in the Department, a total of 86 subjects, were included in this study. Subjects with deficiencies in their muscular system or defective upper extremities or wounds that prevented force application, and professional athletes with hypertrophic muscles, were excluded from the study.

To measure the torques, the implant screwdriver was connected to the digital torquemeter. Then the subjects were asked to wear wet latex gloves (NR Latex, Powdered, NonsterilAmbidextraus)proportional to their hand size and apply torque to the implant screwdriver. The value displayed on the digital screen of the torquemeter (Digital Torque Wrench Lotron, TQ8800, Taiwan) was recorded. After each ten tests, the torquemeter was calibrated with the use of Biomet3itorquemeter.

Data were analyzed with descriptive statistics and Mann-Whitney test and independent t-test using SPSS 21.

## Results

A total of 36 subjects (41.9%) were female and 50 (58.1%) were male. A total of 45 subjects (46.87%) were professors and 41(53.13%) were postgraduate dental students. In relation to age, 50 subjects (58.1%) were in the 25‒34-year age group, 23 (26.7%) were in the 35‒47-year age group and 123 (15.1%) were in the 48‒60-year age group (Table 1).

**Table 1:** The frequencies and percentages of the subjects in terms of gender in the two study groups

**Group**	**Frequency (percentage)**	
	**Female**	**Male**
**Professors**	15 (33.3)	30 (66.7)
**Postgraduate dental students**	21 (51.2)	20 (48.8)

Chi-squared test was used to evaluate the relationship between gender and the study group. The results showed no such a relationship (P=0.093).

Kolmogorov-Smirnov test was used to evaluate the normal distribution of the maximum torque; the results showed normal distribution of this variable. Therefore, the data were parametric (P=0.55). Tables 2 and 3 present the maximum torques in the two study groups.

The results of two-way ANOVA showed that:

**Table 2:** The means and standard deviations of maximum torques in terms of gender in the two study groups

**Group**	**Mean (±SD)**	
	**Female**	**Male**
**Professors**	14.3 (±3)	20.8 (±4.2)
**Postgraduate dental students**	14.7 (±3.4)	18.7 (±4.3)
**Total**	14.5 (±3.2)	19.9 (±4.3)

There were no significant differences in the means of maximum torquesgenerated between the two groups (professors and postgraduate dental students) (P>0.05).

**Table 3:** The means and standard deviations of maximum torques in terms of age groups in the two study groups

**Group**	**Mean (±SD)**		
	25‒34	35‒47	48‒60
**Professors**	14.3 (±4.1)	18.8 (±3.7)	22 (±5.3)
**Postgraduate dental students**	16.4 (±4.3)	21.3 (±1.9)	‒
**Total**	15.9 (±4.1)	19 (±3.6)	22 (±5.3)

**Figure 2:**
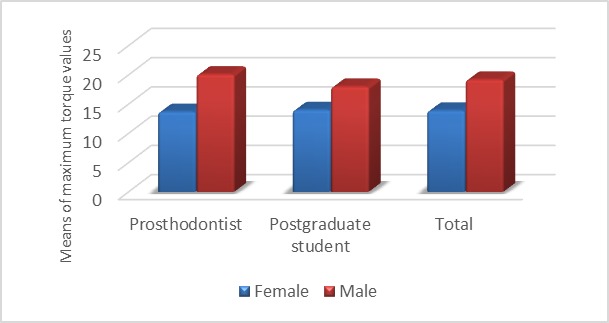
The means of maximum torques in terms of gender.

**Figure 3:**
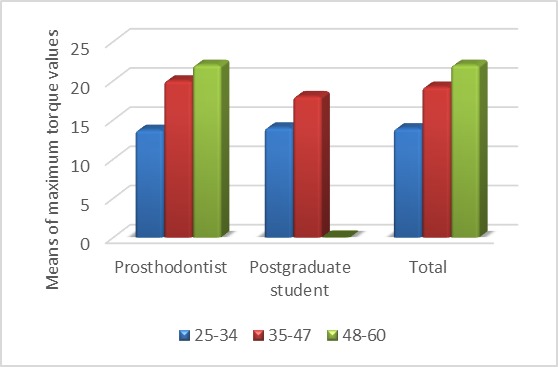
The means of maximum torques in terms of age groups.

1. There was a significant difference in the means of maximum torques produced by males and fe-males, with higher mean maximum torques pro-duced by males (P<0.05; figure 2). There was no reciprocal effect between the study groups and the gender variable, i.e. the effect of gender on the maximum torque in both groups was the same (P>0.05).
2. There were significant differences in the means of maximum torques produced between the dif-ferent age groups (P<0.05; Figure 3). Post hoc Tukey tests were used to determine significant differences between the different age groups. The results of these tests are presented in Tables 1‒3. There were significant differences in the means of maximum torques between the 25‒34-year age group and the two other age groups, with lower maximum torques in the 25‒34-year age group compared to the two other age groups (P<0.05). There was no reciprocal effect be-tween the age groups and the study groups, i.e. the effect of age group of the subjects on the means of maximum torques was the same in both groups (P>0.05).

## Discussion

Abutment screw loosening is one of the most common postoperative complications in implant-supported prosthetic treatments.^15-17^ The prevalence rates of screw loosening in single- and multi-unit restorations have been reported to be up to 12.7% and 6.7%, respectively.^18-21^ Such a problem can pose a major challenge for the clinician, especially in cemented restorations, because in many cases it is not possible to remove the restoration intact; on the other hand, screw loosening can result in the application of extra-axial forces to the implant‒abutment interface, fracturing the screw.^22-24^ During application of tightening torque, the screw increases in length and this tension state created in the screw is referred to as preload. Due to the elastic recoil properties, the screw has a tendency to return to the state without tension, which gives rise to a force that holds the abutment and the screw next to each other. Screw loosening occurs when the forces that have a tendency to separate the components from each other exceed the forces that have a tendency to keep the components next to each other and the preload created within the screw.^25,26^

For example, in a study by Kanawati et al^2^ on 50 dentist sand postgraduate dental students the amount of torque ranged from 11 N/cm to 38 N/cm. A recent study showed that various reasons still prompt some dental practitioners to use hand instruments to tighten abutment screws.^12^ Therefore, if dental practitioners are to use hand instruments without using torque wrenches in different stages of prosthetic procedures of implant treatments, it is necessary for them to be aware of the amount of force they apply to tighten the abutment screw in order to avoid problems associated with the fracture or loosening of the abutment screw.^2,11^

In the present study, the means of maximum torques in male and female professors were 20.8±4.2 and 14.3±3 N/cm, respectively. In addition, the means of maximum torques in male and female postgraduate dental students were 18.7±4.3 and 14.7±3.4 N/cm, respectively. Statistical analyses did not reveal any significant differences in the means of maximum torques between postgraduate dental students and professors.

Nigro et al^24^ evaluated the torque necessary for loosening the screws of two-piece zirconia abutments in dry and wet (artificial saliva) states and reported that the force necessary for loosening abutments whose inner implant threads were contaminated with artificial saliva were significantly higher than those in samples which had been tightened in a dry state. Saliba et al^27^ carried out a study to determine the torque necessary for loosening the abutment screw. In that study the abutment hexagons were removed and titanium cover screws with and without solid lubricant were used. The results showed significantly higher torque necessary for loosening of titanium cover screws with solid lubricant compared to the other type. Guda et al^28^ carried out a study using finite element method (FEM) and showed higher preload in the abutment screw in the environment with the lubricant compared to the dry environment.

Tzenakis et al^29^ showed that repeating the screw tightening procedure in the presence of saliva resulted in higher preload in the prosthetic screws. That study was carried out on screws that tightened prostheses, rather than the abutment tightening screws; in addition, gold screws were used, while at present the majority of tightening screws are made of titanium or its alloys.

In the present study, the mean of the maximum torques in professors was higher than that in postgraduate dental students; however, the difference was not statistically significant. The means of maximum torques in male subjects in both groups and in general were higher than those in female subjects. There was no reciprocal effect between the study groups and the gender variable, i.e. gender had a similar effect on the mean of maximum torques produced in both groups.

The maximum torque in the 25‒34-year age group was less than those in the two other age groups. Age group of the subjects had a similar effect on the means of the maximum torques in both groups (P>0.05).

Contrary to previous studies in which a hand torque meter accurate to 1.5 N/cm was used, in the present study a digital torque meter accurate to 0.1 N/cm was used. Since under loading during the abutment screw tightening was significant, it is suggested that torque meters be used for tightening abutment screws and educational and continuous education programs be held in dental schools.

## Conclusion

In the present study, there were no significant differences in the means of maximum torques produced by professors and postgraduate dental students. The means of maximum torques in males were significantly higher than those in females and gender had no significant effects on the study groups. The maximum torque in the 25‒34-year age group was less than that in the two other age groups.

## Acknowledgments

The authors express special thanks to Ms. Solmaz Maleki Dizaj for her generous support.

## Authors’ contributions

All authors made substantial contributions to the present study. FP, PF contributed to conception and design, acquisition of data, analysis and interpretation of data; they were, moreover, involved in writing and editing the manuscript. FM, SM, VP, JY were the major contributors in preparing and writing the manuscript. All authors have contributed to critical revision of the manuscript, and have read and approved the final version.

## Funding

This study was a port of a thesis and research project (1511) supported and funded by Tabriz University of Medical Sciences.

## Competing interests

The authors declare that they have no competing interests with regards to authorship and/or publication of this work.

## Ethics approval

Not applicable.
